# Editorial: Towards a complex approach to psychotherapy, psychopathology and learning processes: process analysis and evaluation of the clinical efficacy of therapeutic and pedagogical paths

**DOI:** 10.3389/fpsyg.2023.1208575

**Published:** 2023-07-24

**Authors:** Valeria Cioffi

**Affiliations:** ^1^Phenomena Research Group (Italy), Naples, Italy; ^2^Scuola di Specializzazione in Psicoterapia Gestaltica Integrata (SIPGI), Torre Annunziata, Italy

**Keywords:** psychotherapy, pedagogy, education neural networks, patient-therapist relationship, complex systems, graph theory, machine learning

In recent years, there has been growing interest in a more complex and holistic approach to psychotherapy, psychopathology, and learning processes. This approach emphasizes the importance of understanding the interplay between different factors that contribute to psychological distress and the need to develop therapeutic and pedagogical paths that address these factors in a comprehensive and integrated way.

Since psychotherapeutic and educational encounters are complex systems in themselves, the study of these complex systems transcends the mechanistic and reductionist methods used by classical statistics, to describe linear processes, and requires approaches such as complex network theory, neural networks for deep learning, decision trees and cluster analysis methods. These methods, in fact, were born with the aim of simulating the activity of neuronal networks and have proved to be very useful as descriptive means of natural phenomena. They have given a major boost to the field of artificial intelligence research, paving the way for research fields such as computational psychology, psychopathology, and more recently, computational psychotherapy. This complex perspective has made it possible to acquire new knowledge in the fields of psychotherapy, psychopathology and pedagogy and has opened new avenues for interdisciplinary research in these areas.

In this special issue, we bring together a collection of articles that explore the process analysis and evaluation of the clinical efficacy of therapeutic and pedagogical paths in complex cases. Our contributors are leading scholars and practitioners from different fields, including psychology, psychotherapy, education, and neuroscience, who share a common goal of advancing our understanding of how to effectively address the complexities of human experience.

The articles in this special issue cover a wide range of topics, from the analysis of the neural mechanisms underlying psychotherapy to the evaluation of the effectiveness of different pedagogical approaches in diverse settings. They provide theoretical and empirical insights into the different factors that contribute to psychopathology, such as environmental stressors, genetic predispositions, and interpersonal dynamics, and highlight the importance of developing tailored interventions that take these factors into account.

Furthermore, this special issue showcases the latest developments in process analysis and evaluation, including the use of advanced statistical methods and innovative technologies, such as virtual reality, to study the dynamics of therapeutic and pedagogical interactions. By integrating different perspectives and methods, the articles in this issue offer a rich and nuanced understanding of the complexities of psychotherapy, psychopathology, and learning processes, and provide valuable insights into how to develop more effective interventions.

In particular, the first paper “*Babies under 1 year with atypical development: Perspectives of preventive care*.” provides a guide for psychologists dealing with early Autism spectrum disorders interventions and emphasizes the importance of intervening in atypical development in light of typical development (Ferrara et al.).

The second paper is “Rewriting one's own life script: from murder to reintegration into society. In 2016 a two-year program was started by IGAT, Institute of Gestalt and Transactional Analysis based in Naples, in order to support the activities carried out by the NGO ACUDA, a cultural association for re-socialization of convicts and ex-convicts founded in 2001 based in Porto Velho, Rondonia, Brazil, by applying GATES model of psychotherapy to inmates that were participating in the ACUDA project” (Ferrara et al.). This last describes a 2-year program started in 2016 by the IGAT to apply the GATES model of psychotherapy to inmates participating in the ACUDA project in Brazil. The program aimed to help the participants become productive, valuable members of society. At the end of the program, the participants were retested and re-interviewed to assess any changes in their self-perception and their progress toward becoming productive members of society.

The third article, “Unconventional methods in the study of nonpathological personality. A scoping review.”, is a scoping review and is focused on highlighting publications in the literature that have included the use of unconventional methods in the study of non-pathological personality, based on the Big Five theoretical reference model (Mosca et al.).

“Disabilities and migrants: a double educational challenge for an inclusive and plural school.” is the forth article (Tataranni). It discusses the complex and timely issue of school inclusion of foreign children with disabilities in Italy and other parts of the world, especially in light of the increasing number of migrant families with disabled children. The paper highlights the need for multidimensional and integrated approaches to inclusion that incorporate special pedagogy and intercultural pedagogy, as well as cultural anthropology, medical anthropology, ethnopsychiatry, transcultural psychology, and medical sciences. The ultimate aim is to promote a solidary, multicultural, and democratic paideia of the new millennium that supports the social and scholastic inclusion of students who are children of migrants.

The fifth article, “The Gold-Standard Treatment for Social Anxiety Disorder: A Roadmap for the Future”, is a scoping review aimed to summarize, find gaps in the current literature, and formulate future research direction by identifying two broad research questions about the comparative efficacy between in vivo ET and virtual reality exposure therapy (VRET), and the effectiveness of the Pavlovian extinction model in treating SAD (Chowdhury and Khandoker).

The sixth article, “Standard CBT versus integrative and multimodal CBT assisted by virtual reality for generalized anxiety disorder”, is an original research with the aim to compare the efficiency of a standard CBT protocol targeting worries, dysfunctional beliefs, and intolerance of uncertainty with an integrative and multimodal CBT intervention augmented with Virtual Reality (VR). The authors conclude emphasizing the effectiveness of CBT interventions for treating adults with moderate GAD symptomatology and affirming that VR could be integrated within CBT interventions in a single protocol for GAD treatment (Popa et al.).

In conclusion, we believe that this special issue represents an important step toward a more complex and integrated approach to psychotherapy, psychopathology, and learning processes. By bringing together leading scholars and practitioners from different fields, we hope to foster interdisciplinary dialogue and collaboration, and contribute to the development of more effective and tailored interventions that address the complexity of human experience. We hope that this special issue will inspire future research and clinical practice, and help to improve the lives of individuals and communities around the world.

## Author contributions

In this Research Topic, we bring together a collection of articles that explore the process analysis and evaluation of the clinical efficacy of therapeutic and pedagogical paths in complex cases. Our contributors are leading scholars and practitioners from different fields, including psychology, psychotherapy, education, and neuroscience, who share a common goal of advancing our understanding of how to effectively address the complexities of human experience. All authors contributed to the article and approved the submitted version.

